# Acceptability and feasibility of continuous glucose monitoring in people with diabetes: protocol for a mixed-methods systematic review of quantitative and qualitative evidence

**DOI:** 10.1186/s13643-022-02126-9

**Published:** 2022-12-09

**Authors:** Jennifer V. E. Brown, Ramzi Ajjan, Najma Siddiqi, Peter A. Coventry

**Affiliations:** 1 Department of Health Sciences, University of York, York, YO10 5DD UK; 2 Leeds Institute of Cardiovascular and Metabolic Medicine, University of Leeds, Leeds, UK; 3 Hull York Medical School, York, UK; 4 Bradford District Care NHS Foundation Trust, Bradford, UK; 5 York Environmental Sustainability Institute, University of York, York, UK; 6 Leverhulme Centre for Anthropocene Biodiversity, University of York, York, UK

**Keywords:** Continuous glucose monitoring, Diabetes, Mixed methods, Integrative synthesis, Qualitative, Quantitative

## Abstract

**Background:**

Good glycaemic control is a crucial part of diabetes management. Traditional assessment methods, including HbA1c checks and self-monitoring of blood glucose, can be unreliable and inaccurate. Continuous glucose monitoring (CGM) offers a non-invasive and more detailed alternative. Availability of this technology is increasing worldwide. However, there is no current comprehensive evidence on the acceptability and feasibility of these devices. This is a protocol for a mixed-methods systematic review of qualitative and quantitative evidence about acceptability and feasibility of CGM in people with diabetes.

**Methods:**

We will search MEDLINE, Embase, CINAHL, and CENTRAL for qualitative and quantitative evidence about the feasibility and acceptability of CGM in all populations with diabetes (any type) using search terms for “continuous glucose monitoring” and “diabetes”. We will not apply any study-type filters. Searches will be restricted to studies conducted in humans and those published from 2011 onwards. We will not restrict the search by language. Study selection and data extraction will be carried out by two reviewers independently using Rayyan and Eppi-Reviewer, respectively, with disagreements resolved by discussion. Data extraction will include key information about each study, as well as qualitative evidence in the form of participant quotes from primary studies and themes and subthemes based on the authors’ analysis. Quantitative data relating to acceptability and feasibility including data loss, adherence, and quantitative ratings of acceptability will be extracted as means and standard deviations or n/N as appropriate. Qualitative evidence will be analysed using framework analysis informed by the Theoretical Framework of Acceptability. Where possible, quantitative evidence will be combined using random-effects meta-analysis; otherwise, a narrative synthesis will be performed. The most appropriate method for integrating qualitative and quantitative findings will be selected based on the data available.

**Discussion:**

Ongoing assessment of the acceptability of interventions has been identified as crucially important to scale-up and implementation. This review will provide new knowledge with the potential to inform a programme theory of CGM as well as future roll-out to potentially vulnerable populations, including those with severe mental illness.

**Systematic review registration:**

PROSPERO CRD42021255141.

**Supplementary Information:**

The online version contains supplementary material available at 10.1186/s13643-022-02126-9.

## Background

For individuals living with diabetes, glucose control is an important part of self- and clinical management [1–4]. Inadequately controlled glucose levels can lead to serious microvascular and macrovascular complications, creating significant strain on the health system and impairing quality of life [5]. Traditionally, glycaemic control has been assessed using glycated haemoglobin (HbA1c) as a proxy measure of average blood glucose over the 8- to 12-week period prior to measurement [6]. However, due to the nature of HbA1c, this measurement does not detect hypoglycaemia and fluctuations in glucose levels, both of which are implicated in adverse clinical outcome [4, 6]. As any given HbA1c measurement might correspond to a range of mean glucose levels, HbA1c may, for some patients, fail to reliably indicate how well their glucose is controlled [4, 7]. Self-monitoring of blood glucose (SMBG) throughout the day using finger-prick tests has been used as an adjunct to regular HbA1c checks to support glycaemic control [4, 8]. SMBG places the onus on the user, is often perceived as burdensome, and compliance can be low [3, 4, 9–11]. Furthermore, data are not collected continuously during a 24-h period, for example overnight or while the individual is working, driving, or otherwise occupied, leading to glucose levels throughout large parts of the day not being captured [3, 4, 12].

In recent years, continuous glucose monitoring (CGM), measuring glucose in interstitial tissue, has become increasingly used in diabetes care, particularly for individuals with type 1 or insulin-controlled type 2 diabetes [3, 7, 8, 13, 14]. CGM systems provide a more comprehensive assessment of glycaemia by measuring glucose levels every 5–15 min (i.e. 96–298 readings/day), which would be impossible with SMBG [4]. The burden on the individual is also reduced considerably.

CGM systems continue to be developed by a number of different manufacturers (see Lin et al. [15] and Bruttomesso et al. [16], for an overview). They can be categorised into blinded systems (also called professional CGM, where glucose readings are not immediately visible to the wearer), unblinded systems, and flash glucose monitors (also called intermittently scanned CGM or isCGM; see, for example, Wood et al., 2018) [12]. CGM systems work by giving individuals the means to know their blood glucose levels as part of diabetes self-management without controlling blood glucose directly.

While CGM devices have been around for decades, their use has been restricted due to low accuracy, high cost, and bulky devices. More recently, however, CGM sensors made a significant improvement in accuracy and became more affordable, allowing widespread use, particularly for individuals with type 1 diabetes. International guidelines suggest that CGM should be considered as an option to support the assessment of glucose profiles in people receiving insulin, particularly in those having difficulties controlling glucose levels [17]. Different commissioning and prescribing arrangements are in place internationally. For example, in the UK, new guidance from the National Institute for Health and Care Excellence (NICE) is expected to recommend access to CGM or isCGM for all adults with type 1 diabetes [18]. Currently, specific groups of individuals with type 2 diabetes can also access CGM technology, provided they meet the following criteria: insulin treatment plus a learning disability, receiving haemodialysis, or diabetes associated with cystic fibrosis [19]. NICE guidance under development suggests that isCGM be made available to a wider range of adults with type 2 diabetes [20]. Furthermore, there is an emerging body of evidence that CGM may have the potential to improve maternal and infant outcomes in pregnant women with diabetes [21].

Understanding the feasibility and acceptability of innovative health technologies, such as CGM systems, is an important step in assessing their scalability and potential implementation [22, 23]. This also applies to interventions already rolled out, given that better understanding of contextual factors can help address key uncertainties about how and why an intervention does — or does not — work [24]. Continuous and iterative assessment of feasibility and acceptability is promoted by the new Medical Research Council (MRC) framework for the development and evaluation of complex interventions which highlights that uncovering contextual factors and change mechanisms of existing interventions is crucial to intervention improvement and scalability [24]. Importantly, if acceptability and feasibility can be confirmed, there is potential for wider use of CGM in populations who might otherwise struggle to (self-) manage diabetes, such as people with severe mental illness (SMI). An established body of evidence indicates that people with SMI have higher rates of diabetes and die 20 to 25 years younger than the general population [25, 26]. Improved CGM access might improve their glucose profile reducing complication rates, thus addressing this health inequality and mortality gap.

However, there are no recent systematic reviews of the acceptability and feasibility of CGM among people with diabetes. Existing reviews are largely unsystematic without comprehensive searches, clearly defined eligibility criteria, or reproducible methods reported in line with PRISMA (Preferred Reporting Items for Systematic Review and Meta-Analysis) guidance [27]. While available reviews tend to report a positive user experience and possible improvements in quality of life [15, 16, 28, 29], they do not provide robust evidence for acceptability or feasibility. Others have focused on technical parameters and/or effectiveness [12, 30–32], rather than acceptability and feasibility, or included one type of CGM system only [28, 29]. The one existing Cochrane review of CGM is a decade old and focused only on type 1 diabetes; none of the included primary studies reported patient satisfaction or acceptability data, while quality of life was inconsistently reported, and results were inconclusive [33].

This is a protocol for a mixed-methods systematic review of qualitative and quantitative evidence relating to the acceptability and feasibility of CGM. Findings from this review will have the potential to inform understanding of acceptability and feasibility as interlinked concepts that play an important role in the programme theory underpinning the use of CGM in diabetes care.

### Review aim and objective

This systematic review aims to evaluate if CGM systems used to support self-management of diabetes are acceptable and feasible to individuals with diabetes, their carers, and the professionals involved in their care, why (not), and in what context(s).

To address this aim, we will systematically review and synthesise quantitative and qualitative evidence about the acceptability and feasibility of CGM systems to support the self-management of any type of diabetes across a range of populations.

## Methods

This systematic review protocol (PROSPERO registration: CRD42021255141) [34] is reported in line with the PRISMA-P 2015 statement [35], including relevant elements from the adapted PRISMA for reporting systematic reviews of qualitative and quantitative evidence [36]. The completed PRISMA checklist can be found in the online supplementary material.

### Definition of acceptability and feasibility

Within the context of this systematic review, we will broadly consider “acceptability” as a measure of whether people have a satisfactory experience using (service users/carers) or deploying (healthcare professionals) CGM systems (“Do they like it?”). In turn, “feasibility” will be an assessment of the logistical aspects of deploying CGM in clinical practice and/or research settings (“Can it be done?”).

### Outcomes

The primary quantitative outcome of this review will be acceptability of CGM as measured in patient-reported scales. We will include any scale deemed appropriate by the study authors and will standardise means across different questionnaires where appropriate. Secondary outcomes will be feasibility as measured through data on wear time, uptake, and data loss, as well as attrition rates in included studies.

Acknowledging the iterative nature of mixed-methods systematic reviews, additional quantitative outcomes may be explored based on findings from the included qualitative studies. This will be clearly reported.

### Literature searching

We will search MEDLINE, Embase, CINAHL, and CENTRAL for completed and ongoing studies exploring the acceptability and feasibility of CGM in diabetes. We will limit the search to studies conducted with humans. In the interest of producing a current yet comprehensive systematic review, we will limit the search to evidence published in 2011 or later. This will cover important technical developments and advances in accuracy and availability of CGM in the past decade. We will not apply any language or study-type restrictions. Search terms will capture “diabetes” and “continuous glucose monitoring” (including terms for “intermittently scanned CGM” and “flash glucose monitoring”). The full MEDLINE search strategy (developed in partnership with a subject librarian and peer reviewed by an information specialist) is included in Additional file 1.

Thesis databases (Ethos, ProQuest) and conference proceedings of key international diabetes conferences, such as the International Diabetes Federation Annual Conference and the European Association for the Study of Diabetes Annual Meeting, will also be searched. Relevant systematic and narrative reviews will be used for reference searching to identify additional records. Forward citation tracking will be used to find further studies that have cited included papers.

References will be managed and deduplicated in EndNote [37]. The final list of unique search results will be exported into the systematic review app Rayyan [38].

### Study selection

Supported by the machine learning algorithm in Rayyan [38], two reviewers will carry out title and abstract selection independently and in duplicate. Any disagreements will be resolved through discussion.

Full texts will then be sought for all potentially eligible titles and abstracts and imported into EPPI-Reviewer [39], where two reviewers will independently assess eligibility with disagreements again resolved in discussion.

During study selection, publications relating to the same study will be grouped and a main reference identified. If needed, authors will be contacted to confirm related publications.

### Eligibility criteria

Both title and abstract and full-text selection of qualitative and quantitative studies will be based on the following criteria:

#### Population

**Table:** 

Include	Exclude
• Humans, any age, incl. children and adolescents living in the community^a^ • Any diabetes diagnosis (type 1, type 2, diabetes during pregnancy, including gestational diabetes) • Any glycaemic therapies • Any comorbidities, incl. other long-term physical health conditions and any mental health conditions, or no comorbidities • Carers/parents of individuals (any age) with diabetes • Healthcare professionals, including pharmacists, delivering care to individuals (any age) with diabetes	• Animal studies • Continuous glucose monitoring (CGM) used in non-diabetes context, e.g. kidney disease, intensive care (including COVID-19), eating disorders, or monitoring of newborns • Pregnant women during labour/delivery • Inpatients

^a^Studies involving children and/or adolescents and/or their parents, guardians, or other carers will be eligible for inclusion as we expect a considerable part of the existing research to have been conducted in this population. Restricting the eligibility criteria to studies of adults only would risk excluding evidence describing potentially important and relevant experiences of CGM.

Where mixed populations are reported, e.g. participants with and without a diagnosis of diabetes, papers will be included if either results for eligible participants can be extracted separately or the majority of participants (51% or more) were eligible as per the above criteria.

#### Intervention

**Table:** 

*Include*	*Exclude*
• Any kind of CGM system, including blinded, unblinded, and intermittently scanned (flash) systems • CGM systems that are worn on the skin or implanted	• Other methods of glucose monitoring • CGM systems linked to insulin pumps (sensor augmented pumps, artificial pancreas systems, [hybrid] closed-loop systems) • CGM systems tested under extreme circumstances, such as during high-intensity exercise

Specifically, the following categories of CGM systems will be eligible as follows:

1. *Blinded systems (also called professional CGM)*, where the data are stored on the sensor until they are downloaded. No data are fed back to the user automatically. Blinded systems are used primarily in research and as a diagnostic tool in clinical practice [31].
2. *Unblinded systems*, where data are continuously sent to a reading device, such as a specialist reader or a smartphone, allowing the user to observe their blood glucose levels in near-real time. Some systems support the use of alarms or alerts when glucose levels are “out of range”.
3. *Flash glucose monitors* (or isCGM), where the data are collected blinded but can be accessed by the user by scanning the sensor with a smartphone or reader. Both flash and unblinded CGM systems are used primarily by individuals receiving insulin treatment and those with type 1 diabetes to support self-management [31]. Implantable systems fall into this category [40].

#### Comparator

**Table:** 

Include	Exclude
• Self-monitoring of blood glucose • Glycaemic control assessed using only HbA1c checks • Another CGM system • No comparator	• Studies using CGM to evaluate or compare diabetes treatments, such as medication or diet plans

#### Outcomes/data reported

**Table:** 

Include	Exclude
• Quantitative measures of acceptability and feasibility, such as the following: ◦ Completeness of data collection (e.g. percentage of days per week period with data available, percentage of sensors returned) ◦ Patient-reported measures of acceptability, including measures of treatment satisfaction (e.g. questionnaires) ◦ Number (or percentage) of participants declining CGM ◦ Studies stopping early, e.g. due to recruitment issues • Qualitative data relating to the experience of using CGM from a service user, carer, and/or healthcare professional perspective in a research and/or clinical practice context	• Quantitative studies not reporting any feasibility or acceptability data, e.g.: ◦ Studies reporting only clinical diabetes outcomes (e.g. impact on HbA1c) ◦ Studies reporting outcomes like quality of life, diabetes burden, or adverse events that are not direct measures of acceptability (e.g. adverse skin reactions or infections) • Qualitative studies exploring the lived experience of individuals with diabetes, their carers, and/or healthcare professionals more broadly without a direct focus on CGM

#### Study design

**Table:** 

Include	Exclude
• Controlled or uncontrolled studies reporting quantitative data, incl. experimental and cohort designs, e.g. randomised controlled trials (RCTs), non-randomised controlled studies, prospective and retrospective cohort studies with or without a historic or concurrent control group • Studies reporting qualitative data from focus groups, interviews, or written data sources, either as standalone research or as part of a larger (quantitative) study	• Case reports, opinion pieces, including commentaries, editorials, or letters • Systematic and nonsystematic reviews • Economic evaluations • Studies collecting qualitative data but reporting results quantitatively

### Data extraction

Data extraction for all included studies (both quantitative and qualitative) will be carried out in EPPI-Reviewer [39]. Descriptive information will include year of publication, study setting, sample size, participant details (including group [service users, carers/parents, or healthcare professionals], age [for service users], type of diabetes, medication, comorbidities), description of the CGM system used, and relevant outcomes reported.

Quantitative results will be extracted for any direct measures of acceptability such as questionnaires (means and standard deviation or n/N if data were dichotomised by study authors) as well as proxy measures of acceptability and measures of feasibility such as attrition rates and completeness of data collection. We will use an inclusive approach and extract any outcome data broadly relating to the concepts of acceptability and feasibility.

Qualitative data will be extracted in the form of participant quotes reported in included studies as well as interpretative text and overarching themes identified by study authors. To facilitate framework analysis (see below), extraction of qualitative data will be guided by the Theoretical Framework of Acceptability (TFA) [41] and organised into the seven domains of the framework: affective attitude, burden, ethicality, intervention coherence, opportunity costs, perceived effectiveness, and self-efficacy. We will also extract for each paper if the outcomes reported related to prospective, concurrent, or retrospective acceptability, as per the TFA.

All data extraction processes will be piloted on a small number of studies to ensure they are fit for purpose. Changes will be made as necessary. Quantitative data extraction will be carried out by one reviewer, and at least 10% will be checked by another. Qualitative data will be extracted and coded to the framework by one reviewer and then discussed with another.

### Assessment of risk of bias and study quality

As suggested by Noyes et al. [42], the mixed-methods appraisal tool [43] (MMAT) will be used to assess the methodological quality of all included studies. The MMAT includes assessment criteria for qualitative as well as a range of quantitative study designs, offering the ease and convenience of using one tool across all included studies. The following domains are assessed by the MMAT as follows:

- Qualitative studies: Appropriateness of approach used, adequacy of data collection methods, adequacy of findings derived from the data, interpretation of results sufficiently substantiated by data, and coherence between data sources, collection, analysis, and interpretation
- Quantitative studies:
  - ◦ RCTs: Appropriateness of randomisation, comparability of groups at baseline, completeness of outcome data, blinding of outcome assessors, and adherence of participants to assigned intervention
  - ◦ Non-randomised studies: Representativeness of participants, appropriateness of measurements, completeness of outcome data, accounting for confounders, and intervention (or exposure) delivered as intended
  - ◦ Descriptive studies: Relevancy of sampling strategy, representativeness of sample, appropriateness of measurements, risk of nonresponse bias, and appropriateness of analysis
- Mixed-methods studies: Adequacy of rationale for mixed-methods design, effectiveness of integration of study components, adequacy of interpretation of integration, adequacy of addressing discrepancies between components, and methodological quality of study components

Quality assessment will be conducted by one reviewer with at least 10% checked by another.

### Sampling of qualitative studies

Regardless of the number of eligible qualitative studies, we will employ a framework to sample studies for inclusion in the analysis. We will follow methods proposed by Ames et al. [44] which we have successfully deployed previously [45]. The aim will be to sample for maximum variation and data richness, considering study population and relevance to the review question.

1. *Study population*: We will sample all studies that include any of the following study populations:
  - Individuals with type 2 diabetes
  - Individuals with diabetes (any type) and reported comorbid mental and physical health conditions
  - Informal carers
2. *Data richness*: Using the criteria presented below, we will include all studies scoring 4 or higher for data richness. The pool of studies scoring 3 will be scrutinised for any studies offering a unique perspective which will then be sampled. All studies scoring 2 or lower, and not in the populations defined in 1 above, will be excluded from the analysis (Table 1).

**Table 1:** Data richness scoring criteria taken from Ames et al. [44]

Score	Measure
1	Very little qualitative data presented that relate to the synthesis objective. Those findings that are presented are fairly descriptive.
2	Some qualitative data presented that relate to the synthesis objective.
3	A reasonable amount of qualitative data that relate to the synthesis objective.
4	A good amount and depth of qualitative data that relate to the synthesis objective.
5	A large amount and depth of qualitative data that relate in depth to the synthesis objective.

Data richness will be assessed independently by two reviewers. Disagreements will be discussed until consensus is achieved. Details about eligible studies that are not sampled for inclusion in the analysis will be presented in a table along with an explanation of why the study was not sampled.

To avoid the risk of nuanced meaning being lost or biases introduced, we will not attempt translation of qualitative studies where the full text is not available in English and will exclude such papers from the analysis. Articles published in English where the original qualitative data collection was carried out in a non-English language will be eligible for sampling provided translation decisions are reported transparently and in line with the framework proposed by Abfalter et al. [46].

### Certainty of the evidence

To date, no tool exists that specifically supports the assessment of the certainty of evidence in the context of a mixed-methods systematic review. Consequently, separate tools will be used to assess the certainty of the quantitative and qualitative evidence.

For quantitative evidence, we will use the five domains of the GRADE (Grading of Recommendations Assessment, Development and Evaluation) [47] approach to determine if the certainty in the included evidence can be categorised as high, moderate, low, or very low: methodological quality/risk of bias, inconsistency, indirectness, imprecision, and reporting bias.

Correspondingly, CERQual (Confidence in the Evidence from Reviews of Qualitative research) [48] will be used to assess the certainty of the qualitative evidence, as recommended by the Cochrane Qualitative and Implementation Methods Group [49]. Mirroring the GRADE approach, the CERQual domains are as follows: methodological limitations, relevance, coherence, and adequacy of data. The assessment of confidence in the qualitative findings will use four levels (high, moderate, low, very low).

For both qualitative and quantitative evidence, summary of finding tables will be produced, and GRADE/CERQual findings will inform the integrative synthesis.

### Analysis

Given the complexity of “acceptability” as a construct as defined by Sekhon et al. [41], a mixed-methods approach that includes evidence from quantitative and qualitative studies is best suited to answer the research question. The combination and integration of both types of evidence will add value beyond what separate systematic reviews could offer.

We expect to find considerable clinical and methodological heterogeneity in the included studies and will take this into consideration in the analyses to ensure that only evidence from comparable studies is synthesised. We will group the included studies based on key characteristics such as population, type of diabetes, and type of CGM investigated and decide within the team which studies are similar enough to allow a synthesis of their findings.

#### Synthesis of quantitative studies

Where possible, we will synthesise quantitative data, including acceptability questionnaire scores, measures of data completeness, and attrition rates, using random-effects meta-analysis. Attrition rates (n/N of participants “dropped out” or withdrawn) will be summarised to calculate relative risk (and 95% confidence interval) of attrition. The *I*^2^ statistic will be used to estimate statistical heterogeneity [50].

If a meta-analysis of attrition rates is possible, we will further explore the potential impact of study design characteristics on any observed differences in attrition between intervention and control groups. We will conduct sensitivity analyses on attrition outcomes to include only studies with a low risk of attrition bias and compare these findings with all included studies [51].

We will be guided by the Cochrane Handbook in addressing unit of analysis issues [52], for example in cluster-randomised or cross-over trials, as well as in our approach to dealing with missing data [53]. To assess the risk of publication bias within meta-analyses of RCTs, we will generate funnel plots and visually inspect them for asymmetry [54].

In line with recommendations made in the Cochrane Handbook [55], non-randomised studies will be meta-analysed assuming they are deemed to be sufficiently homogenous and at low risk of bias.

Where meta-analysis is not possible, data will be synthesised narratively following methods described by Popay et al. [56] and reported following the synthesis without meta-analysis (SWiM) guidance [57].

#### Synthesis of qualitative evidence

To synthesise data from qualitative studies, we will use the TFA [41] to inform a “best-fit” framework analysis [58], which has been described as “highly suitable for applied … clinical questions in a specific setting or context” [59].

Participant quotes from the primary studies as well as author-inferred themes and interpretations will be mapped against the seven domains of the TFA (affective attitude, burden, ethicality, intervention coherence, opportunity costs, perceived effectiveness, and self-efficacy) with the aim to gain insights into the experience of using or deploying CGM.

Any findings that do not fit the TFA will be analysed separately. We will then explore the potential for any additional concepts derived from the primary studies to further inform or enhance the TFA.

#### Subgroup and sensitivity analyses

Any subgroup analyses will be informed by the qualitative synthesis.

### Integrative synthesis

To maximise the value of this review of quantitative and qualitative evidence, a mixed-methods synthesis will be undertaken. Synthesis will either be conducted following a result-based convergent design, where quantitative and qualitative studies are synthesised separately but simultaneously and then combined in a third synthesis, or a sequential design, where one type of evidence is analysed first with the results from that synthesis and then used to inform the synthesis of the other type of studies [60]. Either synthesis design will allow for an integration of findings that will provide knowledge beyond that which could be gained from separate syntheses alone.

The TFA will be used as the guiding framework throughout all stages of the synthesis.

### Service user and carer involvement

There is strong evidence supporting the involvement of service users and carers in research, and the importance of meaningful involvement is widely recognised [61], including in systematic reviews [62]. Based on findings from the qualitative synthesis, we will identify groups who may face particular challenges when using CGM, for example people with SMI or learning disabilities. Building on existing contacts and networks, we plan to arrange interactive workshops during the integrative synthesis phase to share emergent findings and discuss overlap and discrepancies between qualitative and quantitative evidence. These collaborative sessions will also present an opportunity to use service user perspectives to highlight which areas may need to be addressed in the future to improve the acceptability and feasibility of CGMs for groups who have not yet had a chance to use them.

## Discussion

The proposed review will address a gap in our understanding about acceptability and feasibility of an emerging health technology that has the potential to transform diabetes self-management, including among more vulnerable groups. Understanding if these devices are acceptable and feasible to a range of people, including users, their carers, and healthcare professionals, is a crucial step [24]. It will be the first comprehensive, systematic review in this area and comes at an important time as access to CGM technology is increasing worldwide, including in the UK [18, 20].

By using a truly integrative mixed-methods design to combine qualitative and quantitative evidence, the review findings will offer an in-depth evaluation of the acceptability and feasibility of CGM systems. Data analysis will be informed by the TFA [41], and findings will contribute to our understanding about the overlap of the interrelated concepts of acceptability and feasibility which might have extensions to other applied health research contexts. Findings will have the potential to inform the development of programme theory about the implementation of CGM which may have relevance to individuals living with diabetes and other health conditions, for example SMI. Even though CGM has been in use for several decades, ongoing robust evaluation of feasibility and acceptability is crucial to support reach and scale-up in the context of populations that have hitherto not had access to this technology, including vulnerable adults with SMI. This is recognised and highlighted in the new MRC framework [24]; our systematic review will have the potential to contribute new knowledge to this process.

The inclusion of qualitative evidence of acceptability and feasibility of using CGM in particular will offer a chance to improve understanding about challenges or barriers faced by patient subgroups and whose, as such, experience might be different compared with the general population with diabetes. In particular, individuals with multimorbidity, i.e. several co-occurring mental or physical health problems, might have a uniquely different user experience. There is a body of evidence to suggest that people with SMI engage differently with healthcare systems than the general population [5, 63–66]. A systematic review by Firth et al. [67] including data from over 3000 individuals with psychosis suggests that smartphone ownership is increasing in this population, and that technology-supported self-management is well received. In order to ensure that this vulnerable population with a particularly high burden of diabetes does not miss out on innovative technologies, it is crucial to highlight their lived experience explicitly, where it is appropriate to do so.

Furthermore, draft NICE guidance, due to be published in early 2022, recognises that a wide range of individuals with type 2 diabetes (as well as those with type 1 diabetes) can benefit from access to CGM technology [18, 20]. While people with SMI are not explicitly included in the guidance, they may fall into a number of the categories that are likely to be eligible for CGM technology once the guidance is published: impaired hypoglycaemia awareness, inability to use SMBG, or needing assistance to monitor blood glucose. The draft guidance recognises the importance of education but fails to include specific recommendations for how this should be delivered. Understanding, qualitatively and quantitatively, what does — and does not — make CGM technology feasible and acceptable to use, will be crucial for developing fit-for-purpose education programmes, including for potentially vulnerable populations and those with lower health literacy.

The research question used to inform this draft NICE guidance only relates to effectiveness of CGM; acceptability is not considered [68]. As such, it seems pertinent to produce a robust and comprehensive evaluation of acceptability and feasibility of this technology in parallel with the accelerating roll-out in line with steps for intervention evaluation as recommended by the MRC framework [24].

### Dissemination

The completed review will be submitted for publication in a peer-reviewed journal as well as prepared for presentation at relevant conferences. In addition, existing social media and dissemination channels will be used to reach and engage with a wider audience. A plain language summary will be made available online.

## Supplementary Information

**Additional file 1.** MEDLINE search strategy.

**Additional file 2.** PRISMA-P 2015 Checklist.

## Data Availability

Not applicable.

## Abbreviations

CERQual: Confidence in the Evidence from Reviews of Qualitative research
CGM: Continuous glucose monitoring
GRADE: Grading of Recommendations Assessment, Development and Evaluation
HbA1c: Glycated haemoglobin A1c
isCGM: Intermittently scanned CGM
MMAT: Mixed-methods appraisal tool
MRC: Medical Research Council
NICE: National Institute for Health and Care Excellence (UK)
PRISMA: Preferred Reporting Items for Systematic Review and Meta-Analysis
RCT: Randomised controlled trial
SMBG: Self-monitoring of blood glucose
SMI: Severe mental illness
TFA: Theoretical Framework of Acceptability
