# Supplementary material for: Acceptability and feasibility of continuous glucose monitoring in people with diabetes: protocol for a mixed-methods systematic review of quantitative and qualitative evidence
Source: Syst Rev. 2022 Dec 9;11:263. doi: 10.1186/s13643-022-02126-9 (PMC9733378; doi:10.1186/s13643-022-02126-9)
Supplement: Supplementary file 1 — Additional file 1. MEDLINE search strategy. [file 13643_2022_2126_MOESM1_ESM.docx]

## APPENDIX 1

Ovid MEDLINE(R) ALL <1946 to November 19, 2021>

1 (continu* glucose monitor* or CGM).mp. [mp=title, abstract, original title, name of substance word, subject heading word, floating sub-heading word, keyword heading word, organism supplementary concept word, protocol supplementary concept word, rare disease supplementary concept word, unique identifier, synonyms] 6043

2 flash glucose monitor*.mp. [mp=title, abstract, original title, name of substance word, subject heading word, floating sub-heading word, keyword heading word, organism supplementary concept word, protocol supplementary concept word, rare disease supplementary concept word, unique identifier, synonyms] 404

3 (intermittently scanned glucose monitor* or isCGM).mp. [mp=title, abstract, original title, name of substance word, subject heading word, floating sub-heading word, keyword heading word, organism supplementary concept word, protocol supplementary concept word, rare disease supplementary concept word, unique identifier, synonyms] 54

4 freestyle libre.mp. [mp=title, abstract, original title, name of substance word, subject heading word, floating sub-heading word, keyword heading word, organism supplementary concept word, protocol supplementary concept word, rare disease supplementary concept word, unique identifier, synonyms] 262

5 dexcom.mp. [mp=title, abstract, original title, name of substance word, subject heading word, floating sub-heading word, keyword heading word, organism supplementary concept word, protocol supplementary concept word, rare disease supplementary concept word, unique identifier, synonyms] 251

6 eversense.mp. [mp=title, abstract, original title, name of substance word, subject heading word, floating sub-heading word, keyword heading word, organism supplementary concept word, protocol supplementary concept word, rare disease supplementary concept word, unique identifier, synonyms] 19

7 senseonics.mp. [mp=title, abstract, original title, name of substance word, subject heading word, floating sub-heading word, keyword heading word, organism supplementary concept word, protocol supplementary concept word, rare disease supplementary concept word, unique identifier, synonyms] 8

8 medtronic guardian.mp. [mp=title, abstract, original title, name of substance word, subject heading word, floating sub-heading word, keyword heading word, organism supplementary concept word, protocol supplementary concept word, rare disease supplementary concept word, unique identifier, synonyms] 10

9 blood glucose self-monitoring/is 2061

10 blood glucose self-monitoring/mt 2044

11 1 or 2 or 3 or 4 or 5 or 6 or 7 or 8 or 9 or 10 8533

12 diabet*.mp. [mp=title, abstract, original title, name of substance word, subject heading word, floating sub-heading word, keyword heading word, organism supplementary concept word, protocol supplementary concept word, rare disease supplementary concept word, unique identifier, synonyms] 761084

13 exp Diabetes Mellitus/ 461230

14 hyperglyc?emia.mp. [mp=title, abstract, original title, name of substance word, subject heading word, floating sub-heading word, keyword heading word, organism supplementary concept word, protocol supplementary concept word, rare disease supplementary concept word, unique identifier, synonyms] 69957

15 exp Hyperglycemia/ 38436

16 hypoglyc?emia.mp. [mp=title, abstract, original title, name of substance word, subject heading word, floating sub-heading word, keyword heading word, organism supplementary concept word, protocol supplementary concept word, rare disease supplementary concept word, unique identifier, synonyms] 52116

17 exp Hypoglycemia/ 29765

18 12 or 13 or 14 or 15 or 16 or 17 811101

19 11 and 18 7213

20 exp animals/ not humans/ 4917379

21 19 not 20 7089

22 limit 21 to yr="2011 -Current" 5435
